# The Changes in Mitochondrial Morphology and Physiology Accompanying Apoptosis in *Galleria mellonella* (Lepidoptera) Immunocompetent Cells during *Conidiobolus coronatus* (Entomophthorales) Infection

**DOI:** 10.3390/ijms241210169

**Published:** 2023-06-15

**Authors:** Agata Kaczmarek, Anna Katarzyna Wrońska, Mieczysława Irena Boguś

**Affiliations:** 1 Museum and Institute of Zoology, Polish Academy of Science, Wilcza 64, 00-679 Warsaw, Poland; 2 Witold Stefański Institute of Parasitology, Polish Academy of Sciences, Twarda 51/55, 00-875 Warsaw, Poland; 3 Biomibo, 04-872 Warsaw, Poland

**Keywords:** aerobic and anaerobic respiration, ATP, calcium ions, caspase-9, cytochrome c, megachannels, membrane potential, mitochondria, oxygen consumption

## Abstract

Mitochondria have been shown to play an important role in apoptosis using mammalian cell lines. However, their role in insects is not fully understood; thus, more indepth studies of insect cell apoptosis are necessary. The present study investigates mitochondrial involvement during *Conidiobolus coronatus*-induced apoptosis in *Galleria mellonella* hemocytes. Previous research has shown that fungal infection could induce apoptosis in insect hemocytes. Our findings indicate that mitochondria undergo several morphological and physiological changes during fungal infection, e.g., loss of mitochondrial membrane potential, megachannel formation, disturbances in intracellular respiration, increased nonrespiratory oxygen consumption in mitochondria, decreased ATP-coupled oxygen consumption and increased non-ATP–coupled oxygen consumption, decreased extracellular and intracellular oxygen consumption, and increased extracellular pH. Our findings confirm that *G. mellonella* immunocompetent cells demonstrate Ca^2+^ overload in mitochondria, translocation of cytochrome c-like protein from mitochondrial to cytosol fraction, and higher activation of caspase-9-like protein after *C. coronatus* infection. Most importantly, several of the changes observed in insect mitochondria are similar to those accompanying apoptosis in mammalian cells, suggesting that the process is evolutionarily conserved.

## 1. Introduction

Despite causing the loss of cells, host cell death may be an important defence mechanism occurring in response to microbial infection. Indeed, apoptotic and regulated necrotic processes such as necroptosis, pyroptosis, and extracellular trap-related cell death-ETosis, can have a significant influence on the outcome of a microbial insult [1]. Although most studies of programmed cell death have examined the result of viral and bacterial infections, evidence suggests that apoptosis and regulated necrosis processes also play a key role in the interplay between pathogenic fungi and host mammalian cells [2].

Apoptosis is a highly complex form of programmed cell death involving an energy-dependent cascade of molecular and cellular events. It represents a vital part of the immune response to pathogens, which leads to the destruction of the intracellular niche of microbial replication. Furthermore, the elimination of pathogen-containing apoptotic bodies by secondary phagocytes and the presentation of antigens derived from the apoptotic material by dendritic cells represent important antimicrobial effector mechanisms in mammals [3]. Pathogenic fungi have, therefore, evolved multiple distinct mechanisms for modulating host-cell apoptosis.

Apoptosis occurs via extrinsic and intrinsic signalling pathways. The former involves the activation of the Fas and Tumour Necrosis Factor (TNF) death receptors; in contrast, the intrinsic pathway generates intracellular signals through the host mitochondria [4] and all inducers of intrinsic apoptosis trigger the mitochondria [5]. The early stages of programmed cell death are characterised by the disruption of mitochondrial function and changes in its membrane potential, a central feature of mitochondrial health, as well as alterations to its oxidation-reduction potential. Inner mitochondrial membrane potential has a key influence on calcium ion (Ca^2+^) uptake and storage, reactive oxygen species (ROS) generation and detoxification and, most importantly, the synthesis of adenosine triphosphate (ATP) by oxidative phosphorylation [6]. Therefore, the degree of membrane depolarization is a good indicator of mitochondrial dysfunction, which is increasingly implicated in toxicity [7]. Changes in membrane potential, along with a decreased ATP to ADP ratio, increased mitochondrial matrix calcium levels, oxidative stress, and release of cytochrome c into the cytosol are all presumed to be associated with a transition in mitochondrial permeability; such changes result in the disruption of ions and small molecule homeostasis via the mitochondrial permeability transition pore (MPTP) [8]. Cytochrome c, an important proapoptotic protein released from mitochondria, binds apoptotic peptidase activating factor 1 (Apaf-1) and procaspase-9 to form the apoptosome complex. Once formed, the apoptosome activates procaspase-3 [5].

Although fungal pathogens (e.g., *Candida albicans*, *Cryptococcus neoformans*, and *Aspergillus fumigatus*) are known to induce or manipulate apoptosis in both immune and nonimmune cells in mammalian models [9,10,11,12,13,14,15,16], this relationship is not well understood in insects. With the recent growth of interest in the replacement of mammalian research models with insect ones in preliminary studies and the important role played by apoptosis during fungal infection, there is a growing need to better understand the effect of fungal infection on programmed cell death in insects.

Most studies on apoptotic regulation in insects have been carried out using the *Drosophila* system. Although apoptotic events are evolutionarily conserved, some fundamental differences between the apoptotic signalling taking place in *Drosophila* and mammals have been reported. These differences relate to the role of the mitochondria in the apoptosis process. While a number of these studies have shown that mitochondrial factors may not be involved in *Drosophila* apoptosis, certain recent reports have provided contrasting evidence, suggesting that mitochondrial factors may play a possible role during apoptosis in *Drosophila* [17,18]. In Lepidoptera, the mitochondrial mechanisms of apoptosis induction, including the regulation of cytochrome-*c* release and the dependence of downstream activation of caspases, have been well described in the Sf9 cell line, *Bombyx mori*, and *Spodoptera litura* [19,20,21,22,23].

While the role of apoptosis during a fungal infection in insects remains unclear, some research has described the presence of apoptosis and caspase-like proteins activation [24,25] and oxidative stress [26] in immunocompetent cells of *Galleria mellonella* during *Conidiobolus coronatus* infection. *C. coronatus*, a cosmopolitan soil fungus, is an opportunistic pathogen which causes fungal infection (conidiobolomycosis) in humans and other mammals [27,28,29]. Initially, cases of conidiobolomycosis were detected in areas with tropical and subtropical climates; however, due to climate change and modern advances in global travel, the disease now can spread in temperate climates [30,31,32,33]. Therefore, there is a need to develop more and more efficient systems to test fungal activity and the process of infection.

Over recent years, interest has grown in replacing mammalian models with those based on insect systems for biological and commercial research. A number of close structural and functional similarities have been found between the innate immune systems of mammals and the insect immune response. As such the idea of using *G. mellonella* as a model organism in conidiobolomycosis is an attractive one that merits further attention. *C. coronatus* has selective entomopathogenic activity [34] and was found to induce approximately 90% mortality in *G. mellonella* last instar larvae populations [35,36,37]. *G. mellonella* larvae have been used for evaluating the virulence of fungal pathogens in humans and for assessing the efficacy of antifungal agents [38,39,40,41]. Compared to mammalian models, larvae have considerable advantages: they are cost effective, easy to rear, and allow time-efficient drug discovery and physiological process research [42].

The main aim of the present work was to determine the effect of *C. coronatus* infection on the morphology and physiology of mitochondria as part of the apoptosis process in *G. mellonella* hemocytes. The testable hypothesis was that exposure to entomopathogenic fungus caused the cell death of insect hemocytes by apoptosis via the intrinsic pathway, i.e., influencing mitochondrial activity and morphology.

## 2. Results

### 2.1. The Changes in Mitochondrial Activity in G. mellonella Hemocytes after Fungal Infection

**Figure 1:**
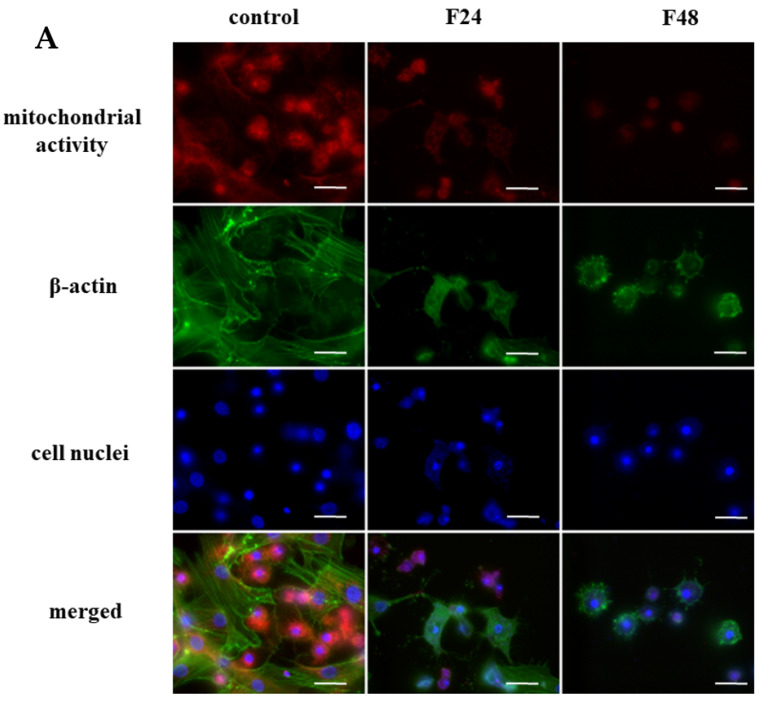

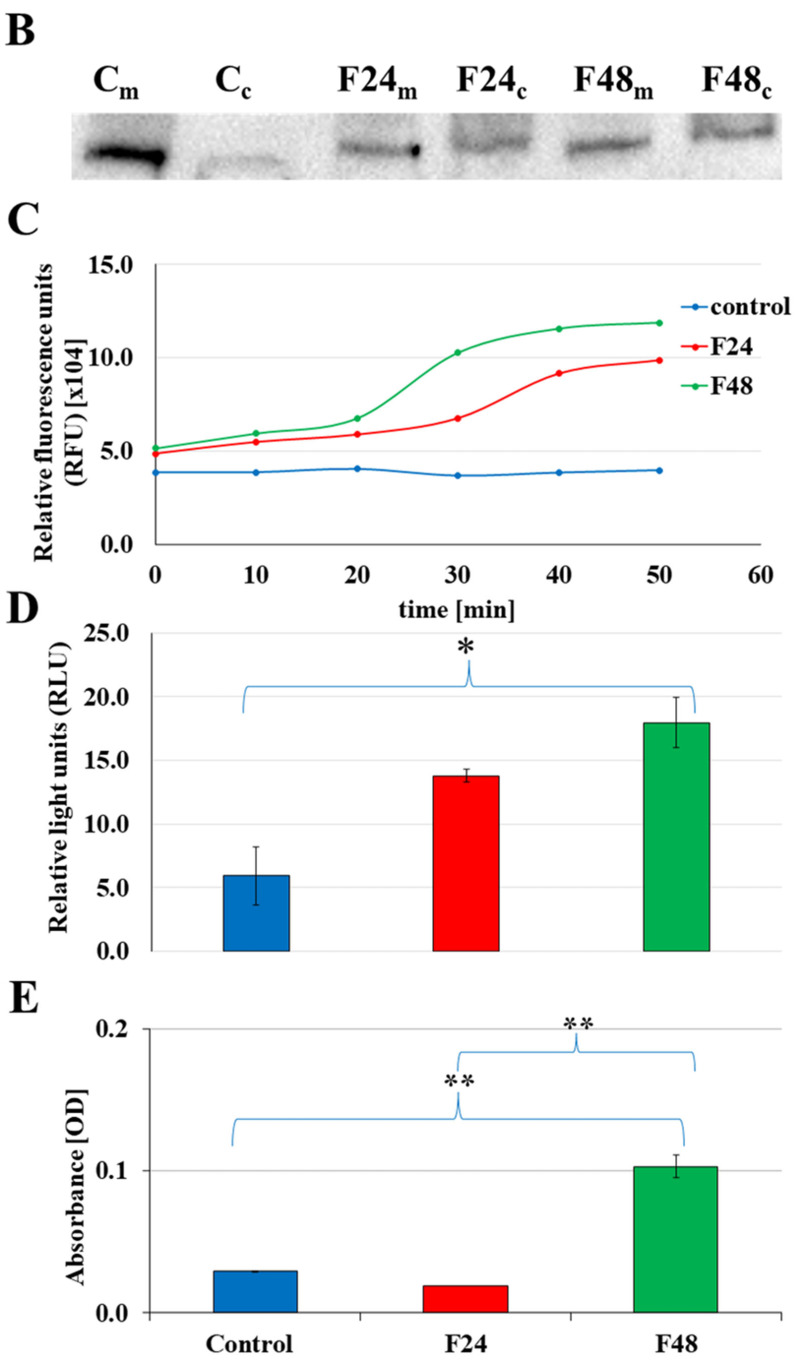
The changes in mitochondrial activity in *G. mellonella* hemocytes after fungal infection. (**A**) The detection of mitochondrial activity in *G. mellonella* hemocytes after fungal infection by fluorescence microscopy; (**B**) Detection of cytochrome c-like protein translocation from the mitochondrion to the cytosol fraction using Western blot; (**C**) Time-dependent changes in calcium levels in *G. mellonella* hemocytes after fungal infection. (**D**) Changes in ADP/ATP ratio in *G. mellonella* hemocytes after fungal infection. (**E**) Changes in caspase-9-like protein activity in *G. mellonella* hemocytes after fungal infection; F24 larvae sampled immediately after 24-h exposure to fungal infection; F48 larvae sampled 24 h after 24-h exposure; m—mitochondrial fraction; c—cytosol fraction; * *p* ≤ 0.05, ** *p* < 0.001 (*t*-Student test); the experiments were conducted in three repeats; scale bar—25 µm.

The activity of mitochondria was measured by fluorescence microscopy using the MITO-ID Red Detection Kit (GFP-CERTIFIED^®^). The distribution of mitochondria in hemocytes of *G. mellonella* is presented in Figure 1A. In the controls, high red fluorescence (mitochondrial activity) was observed in both cell types; they are visible as single red-stained points in the cytoplasm in plasamtocytes and as bright red fluorescent circles around the cell nuclei in granulocytes. After fungal infection, considerable disintegration of hemocytes was observed, together with a lower intensity of red fluorescence. This lower red fluorescence was accompanied by lower green fluorescence (actin staining) in the FITC channels.

### 2.2. The Translocation of Cytochrome c-like Protein from Mitochondrial to Cytosol Fraction in G. mellonella Hemocytes during Fungal Infection

The changes of concentration of mitochondrial and cytosol fraction containing the cytochrome c-like protein in the *G. mellonella* hemocyte after fungal infection are presented in Table 1. According to the presented research, the *C. coronatus* infection caused the decrease in mitochondrial and an increase in cytosol fraction of protein concentration in the insect hemocyte.

During fungal infection, cytochrome c-like protein was present in both the mitochondrial and cytosol fractions in *G. mellonella* hemocytes, as detected by Western blot (Figure 1B). In the control cells, cytochrome c-like protein was only observed in the mitochondrial fraction. Our findings indicate that translocation of cytochrome c-like protein occurs during infection.

### 2.3. The Flux of Ca^2+^ Level in G. mellonella Hemocytes after Fungal Infection

The changes in Ca^2+^ levels in cells were measured by spectrofluorometric analysis using the FLUOFORTE Calcium Assay Kit. The time-dependent changes in Ca^2+^ level in *G. mellonella* hemocytes after fungal infection are shown in Figure 1C. An increase in calcium level was observed in samples F24 and F48 just after the fluorescence readings were started; at the last time point (50 min), the relative fluorescence units (RFU) were 2.5 times higher than in control cells for F24 and 3.01 times higher for F48.

### 2.4. Detection of Changes in ADP/ATP Ratio in G. mellonella Hemocytes after Fungal Infection

The ADP/ATP ratio assay kit was used for measuring ADP and ATP levels in *G. mellonella* hemocytes during *C. coronatus* infection. After fungal infection, an increase in the ADP/ATP ratio was detected (Figure 1D), reaching 5.95 in control cells, 13.80 in the F24 group, and 17.99 in the F48 group; ANOVA, Tukey’s HSD Test; F(2,3) = 11.84, *p* = 0.04, MS = 6.31, df = 3.00).

### 2.5. Changes in Caspase-9-like Protein Activity in G. mellonella Hemocytes after Fungal Infection

The activity of the caspase-9-like protein in wax-moth hemocytes is shown in Figure 1E. After fungal infection, caspase-9-like protein level was found to fluctuate. A slight decrease in activity was detected after 24 h of infection (F24) (1.6-times lower than in the control); however, this difference was not statistically significant. In contrast, activity increased 3.5-fold when compared with untreated larvae after 48 h (F48); ANOVA, Tukey’s HSD Test: F(2,6) = 160.08, *p* < 0.001, MS < 0.001, df = 6.00).

### 2.6. Changes in Membrane Potential in G. mellonella Hemocytes after Fungal Infection

**Figure 2:**
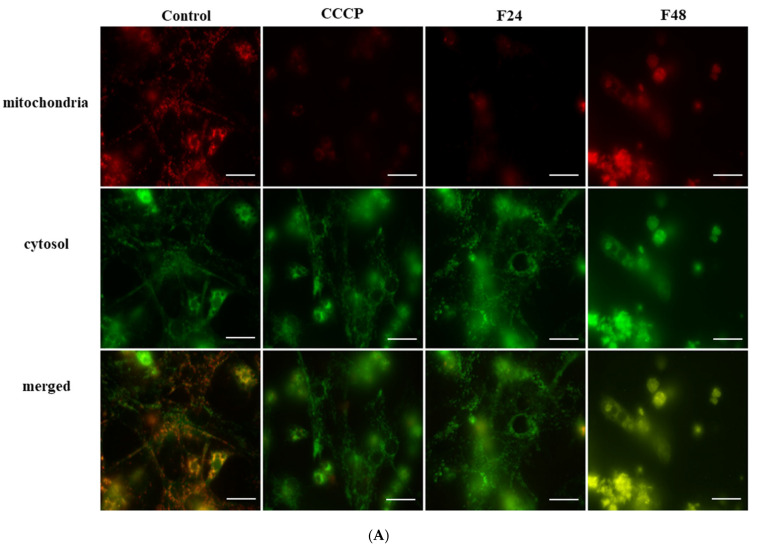

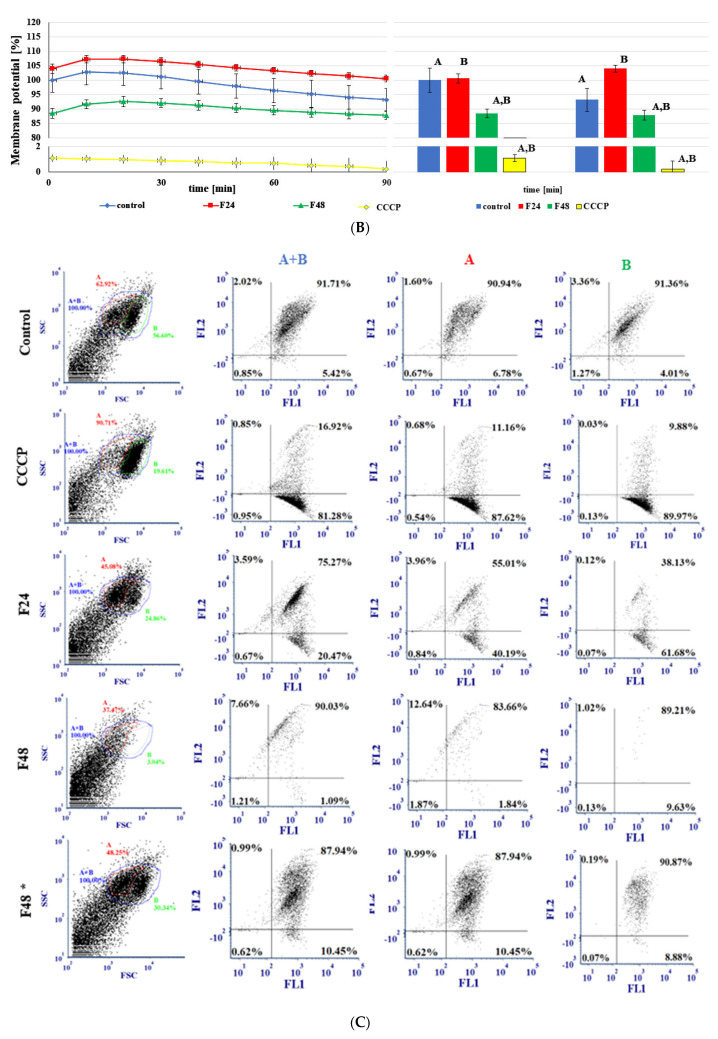
The changes in membrane potential in *G. mellonella* hemocytes after fungal infection (**A**) fluorescent microscopy analysis; scale bar—25 µm. (**B**) spectrofluorometric measurement; the statistical significance is marked with the same letters (A, B; ANOVA, the Turkey post hoc test), the experiments were conducted in three repeats; (**C**) flow cytometry analysis. F24 larvae sampled immediately after 24-h exposure to fungal infection; F48 larvae sampled 24 h after 24-h exposure; F48* larvae sampled 24 h after 24-h exposure with no sign of infection; carbonyl cyanide 3-chlorophenylhydrazone (CCCP)—positive control; A—population A; B—population B; A + B—both populations, FL1—FITC channel FL2—Texas Red channel; the experiments were conducted in three repeats.

The MITO-ID^®^ Membrane Potential Kit measures fluctuations in mitochondrial membrane potential (MMP) based on a cationic dual-emission dye that exists as green fluorescent monomers in the cytosol and accumulates as red fluorescent J-aggregates in the mitochondria. Mitochondria having a low membrane potential will accumulate low concentrations of dye and thus will exhibit green fluorescence, while more highly polarized mitochondria will exhibit red fluorescence. Cells exhibit a shift from red to green fluorescence as the mitochondrial function becomes increasingly compromised. The changes in membrane potential are shown in Figure 2.

The changes in membrane potential identified by fluorescence microscopy are shown in Figure 2A. The untreated cells demonstrated high-intensity green and red fluorescence. In contrast, hemocytes treated with CCCP and those from infected insects displayed at the low intensity of red fluorescence; this reflects a shift from red to green fluorescence, indicating changes in membrane potential.

Spectrofluorometric analysis (Figure 2B) confirmed an increase in membrane potential in *G. mellonella* hemocytes from the F24 group, as well as a decrease in hemocytes from the F48 group. Membrane potential was 100 ± 4.24% value for control cells in the first minute, 103.97 ± 1.65% for F24, and 88.49 ± 1.69% for F48 (ANOVA, Tukey’s HSD Test, F(3,11) = 884.43, *p* = 0.00, MS = 8.60, df = 11.00). After 90 min of measurement, a decrease in membrane potential was observed in all types of cells (ANOVA, Tukey’s HSD Test, F(3,12) = 523.58, *p* = 0.00, MS = 14.03, df = 12.00): controls (93.19 ± 3.92%), F24 (100.56 ± 1.15%), and F48 (87.84 ± 1.54%).

The changes in membrane potential detected in flow cytometry are presented in Figure 2C. After flow cytometry analysis, two populations of *G. mellonella* hemocytes were observed (described as A and B). In the control samples, both populations (A + B) can be seen in the double-positive top-right quadrant, as well as each individual population (A or B), indicating that both red and green fluorescence was observed in hemocytes. After CCCP treatment, and in hemocytes from infected insects, the changes were mostly detected in the B population, where more cells are placed solely in the single positive quadrant (FITC), this change indicates shifts from red to green fluorescence. In addition, the B population had disappeared during fungal infection, reflecting the death of hemocytes from this population. What is more interesting is that similar changes were not observed in hemocytes taken from fungus-treated larvae without any sign of infection (F48*). In this case, both populations’ cells have similar changes similar to this observed in the control.

### 2.7. Detection of Mitochondrial Permeability Transition Pore (MPTP) Opening in G. mellonella Hemocytes after Fungal Infection

The mitochondrial permeability transition pore (MPT pore or MPTP) is a nonspecific channel formed by components of the inner and outer mitochondrial membranes and appears to be involved in the release of mitochondrial components during cell death. Although MPTPs alternate between open and closed states in healthy cells, they dramatically alter the permeability of the mitochondria during cell death.

**Figure 3:**
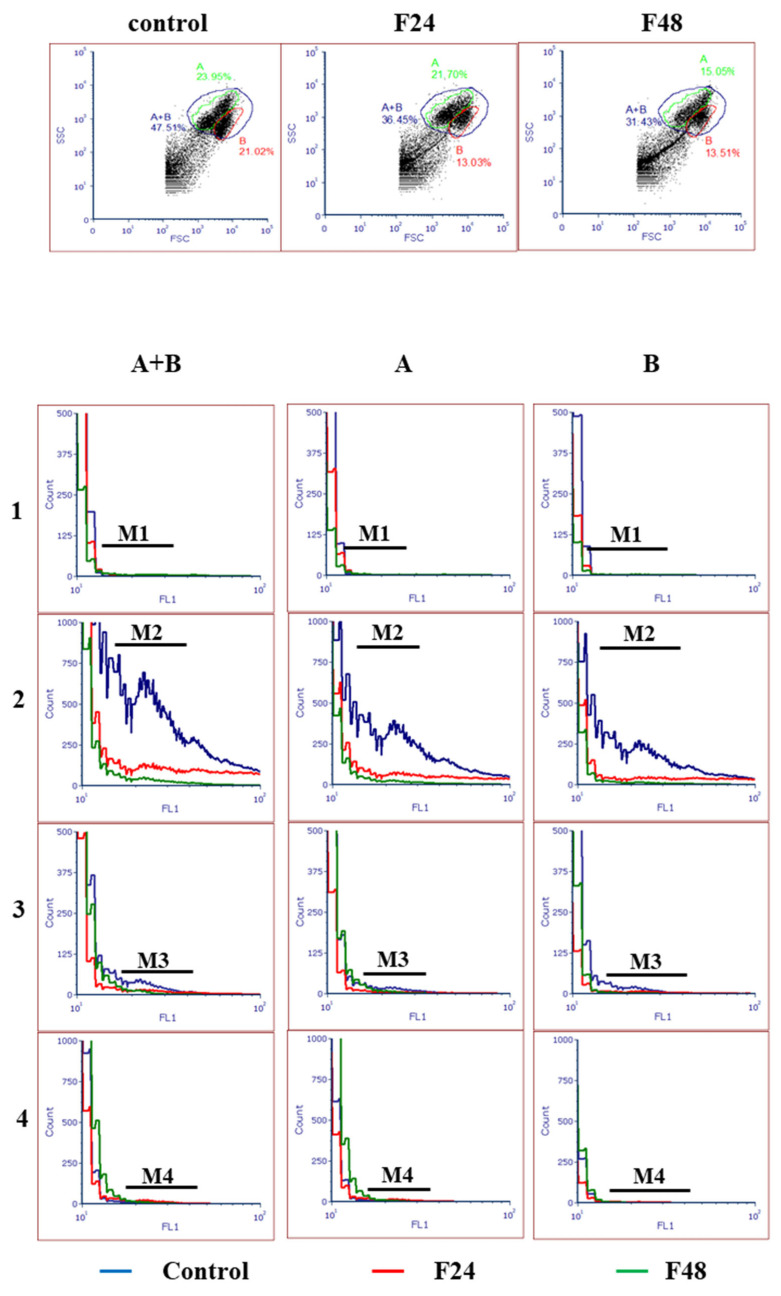
Mitochondrial permeability transition pore (MPTP) opening in *G. mellonella* hemocytes after fungal infection. 1—cells without staining (M1); 2—Samples stained with MPTP Staining Dye (M2, fluorescence signal from both cytoplasm and mitochondria); 3—Samples stained with MPTP Staining Dye and treated with cobalt(II) chloride (CoCl_2_; M3, mitochondrial fluorescence); 4—Samples stained with MPTP Staining Dye and treated with CoCl_2_ and Ionomycin (M4); F24 larvae sampled immediately after 24-h exposure to fungal infection; F48 larvae sampled 24 h after 24-h exposure; A—population A; B—population B; A + —both population; FL1-FITC channel; the experiments were conducted in three repeats.

The hemocytes from fungal-treated and control insects were analyzed by flow cytometry (Figure 3). Tube 1 (M1) included unstained samples, used for instrument setup. Samples stained with MPTP staining dye (M2) showed cumulative fluorescence from both the cytoplasm and mitochondria. The tubes treated with MPTP staining dye and CoCl_2_ (M3) only demonstrated mitochondrial fluorescence. The tubes treated with all reagents (M4) showed the lowest fluorescence. The difference in fluorescence between M3 and M4 indicates the degree of MPTP activation and subsequent depolarization of the mitochondrial membrane. Our findings indicate a higher intensity of fluorescence in both the cytoplasm and mitochondria (M3) in the control cells than in F24 and F48, which might indicate a higher level of hemocyte mortality after fungal infection. Moreover, small differences were found between M3 and M4 in hemocytes from fungus-treated insects, which indicates the opening of MPTP in *G. mellonella* hemocytes (in both A and B populations) after *C. coronatus* infection.

### 2.8. The Changes in Oxygen Consumption in G. mellonella Hemocytes after Fungal Infection

**Figure 4:**
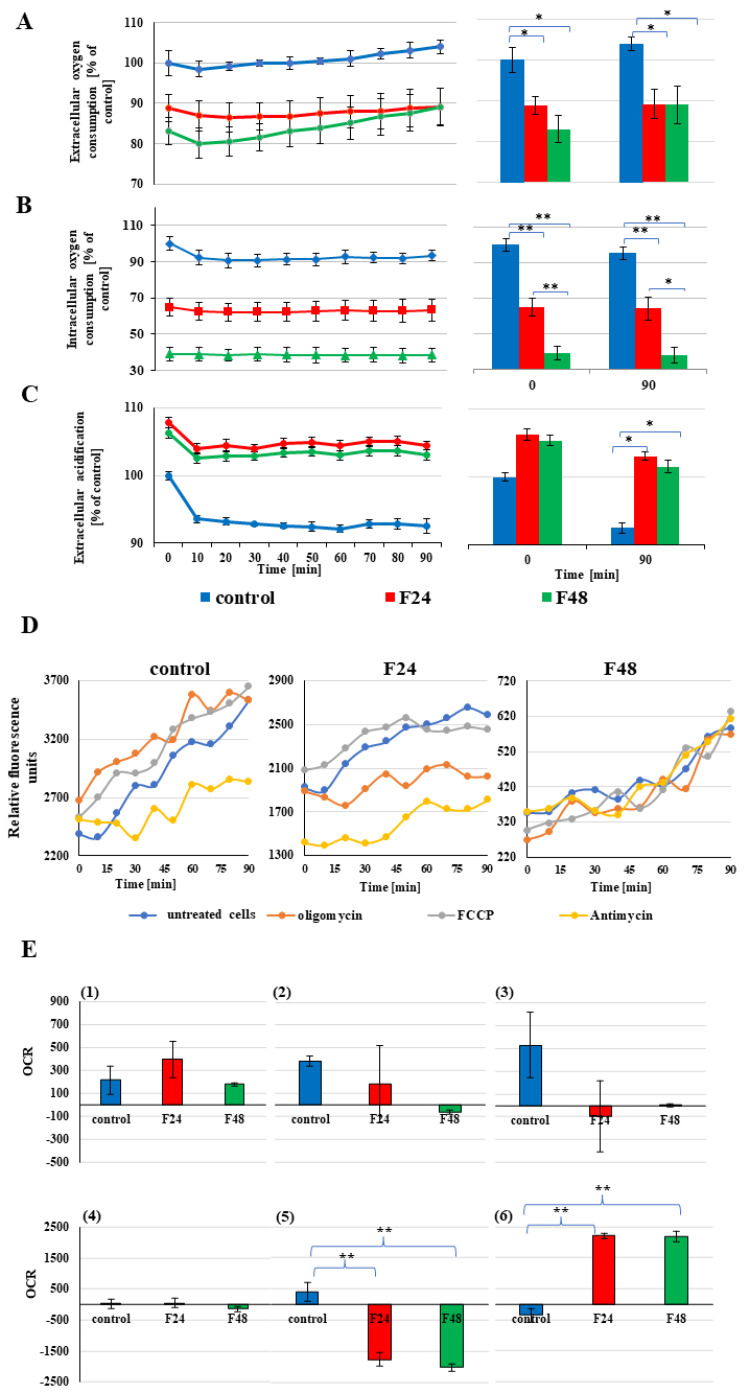
Oxygen consumption in *G. mellonella* hemocytes after fungal infection. (**A**) Extracellular oxygen consumption in *G. mellonella* hemocytes after fungal infection; (**B**) Intracellular oxygen consumption in *G. mellonella* hemocytes after fungal infection; (**C**) Extracellular acidification in *G. mellonella* hemocytes after fungal infection; (**D**) Signal profiles of hemocyte oxygen consumption obtained from control and infected insects; (**E**) Characterization of mitochondrial function of hemocytes obtained from control and fungus treated insect: (**1**) nonrespiratory oxygen consumption; (**2**) based respiratory rate; (**3**) maximal respiratory rate; (**4**) spare respiratory capacity; (**5**) ATP-couples oxygen consumption; (**6**) non-ATP-coupled oxygen consumption; carbonyl cyanide-p-trifluoromethoxyphenylhydrazone (FCCP); OCR—oxygen consumption rate; F24 larvae sampled immediately after 24-h exposure to fungal infection; F48 larvae sampled 24 h after 24-h exposure; * *p* < 0.05, ** *p* < 0.001 (ANOVA, the Turkey post hoc test), the experiments were conducted in three repeats.

The changes in oxygen consumption observed in *G. mellonella* hemocytes after fungal infection are presented in Figure 4.

Our data indicates that *C. coronatus* infection caused a decrease in both extracellular and intracellular oxygen consumption together with an increase in glycolytic flux. This indicates that extracellular acidification increased in the *G. mellonella* hemocytes (Figure 4A–C).

After fungal infection, a decrease in extracellular oxygen consumption (Figure 4A) was detected. Oxygen consumption was estimated as 100 ± 3.03% in the cells taking from control insect, 88.04 ± 2.15% for F24, and 88.06 ± 3.34% for F48 in the first minute of measurement (ANOVA, Tukey’s HSD Test, F(2,5) = 15.67, *p* = 0.01, MS = 14.08, df = 5.00).

A similar result was obtained for intracellular oxygen consumption (Figure 4B). The mean fluorescence was calculated as 100 ± 3.73% for controls in the first minute of measurement. These levels fell to 64.92 ± 4.96% for F24 and 39.08 ± 3.66% for F48 (ANOVA, Tukey’s HSD Test: F(2,5) = 78.48, *p* < 0.001, MS = 28.40, df = 5.00).

Fungal infection caused an increase in extracellular acidification (Figure 4C, F(2,5) = 52.14, *p* < 0.001, MS = 34.73, df = 5.00). In hemocytes from the infected insects, the level of acidification was 106.23 ± 0.81% for F24, compared to the control cells, and 105.40 ± 0.78% for F48.

The fluorescence profiles reflecting hemocyte oxygen consumption in the control and infected insects are presented in Figure 4D. Fungal infection was associated with a lower RFU value in both the F24 and F48 probes compared with controls, indicating a decrease in all oxygen-consumption parameters, viz. ATP-coupled respiration (oligomycin treatment), maximal respiration (FACP treatment) and nonrespiratory oxygen consumption (actinomycin A treatment). Moreover, during infection, nonrespiratory oxygen consumption is more favoured in hemocytes compared to maximal respiration ATP-coupled respiration. A significant increase was noted in nonrespiratory ATP consumption (ANOVA, Tukey’s HSD Test, F(2,6) = 161.05, *p* < 0.001, MS = 40,390.00, df = 6.00) together with a decrease in ATP-coupled oxygen consumption (ANOVA, Tukey’s HSD Test, F(2,6) = 114.86, *p* < 0.001, MS = 41,179.00, df = 6.00). Detailed changes in oxygen consumption are presented in Figure 4E.

## 3. Discussion

Our previous research has highlighted the important role of apoptosis, oxidative stress, and caspases 1-9-like protein activation in *G. mellonella* hemocyte destruction during *C. coronatus* infection [24,25,26] with greater activation of caspase 9-like protein than caspase 8-like protein in *G. mellonella* hemocytes [25]; this might suggest that the intrinsic pathway plays the dominant role in apoptosis activation in insect immunocompetent cells during *C. coronatus* infection.

Our present findings also confirm that the initiator of apoptosis, caspase-9-like protein (Figure 1E), demonstrates higher activity in wax-moth hemocytes taken from insects treated with fungus. The literature data also confirm the presence of the homologue of mammalian caspase-9 in Lepidoptera (Lep-caspase-5) [43]. In *S. litura*, this protein (*Sl*-caspase-5) can cleave *Sl*-procaspase-1, a homologue of mammalian caspase-3, which directly causes apoptosis [44]. Our data indicate that a similar protein, demonstrating higher activity towards apoptosis and fungal infection, is also present in *G. mellonella*; however, more detailed research is needed to compare the structure and physiology of detected protein with mammalian caspase-9.

Such elevated caspase-9-like protein activity suggests that the intrinsic pathway might play an important role in apoptosis activation, and thus hemocyte destruction, during *C. coronatus* infection. This pathway is closely connected with the activity of mitochondria [45]. Our present findings indicate that *C. coronatus* infection resulted in the changes in mitochondria morphology and activity in *G. mellonella* hemocytes. Mitochondria are double membrane-bound organelles found in most eukaryotic organisms. They act as prominent energy carriers and ATP production centres [46], and influence the immune response [47,48]. Mitochondria are an important source of ROS within most mammalian cells [49,50,51]. While ROS production contributes to mitochondrial damage in a range of pathologies, it is also a key component in redox signalling from the organelle to the rest of the cell [52,53].

Mitochondria have been found to play a crucial role in apoptosis activation in an insect cell line (Sf-9) after azadirachtin (insecticidal tetranortriterpenoid) treatment [20]. Our observations confirm that several physiological and morphological changes occur in mitochondria during fungal infection and underline their pivotal role in insect death after *C. coronatus* treatment. They also highlight the similarities in these processes between insects and mammals.

In mammal cells, mitochondrial functions are influenced by Ca^2+^ overload [54]. Similarly, higher levels of Ca^2+^ were also detected in *G. mellonella* hemocytes after fungal infection in the present study (Figure 1C). In mammals, the endoplasmic reticulum (ER) transmits Ca^2+^ signals to the mitochondria, which decode them into specific inputs to regulate essential functions, including metabolism, energy production, and apoptosis [55]. Under physiological conditions, the accumulation of Ca^2+^ in mitochondria stimulates oxidative metabolism through the modulation of Ca^2+^-sensitive dehydrogenases and metabolite carriers [56,57]. During apoptosis activation via intrinsic pathways in mammals, Ca^2+^ acts as a critical sensitizing signal; the Ca^2+^ level can be seen to increase at both the early and late stages of the apoptotic pathway and two apoptogenic mechanisms have been proposed: Ca^2+^ release from the ER, and capacitive Ca^2+^ influx through Ca^2+^ release-activated Ca^2+^ channels [58,59,60,61]. Taking into account the evolutionary conservatism of programmed cell death signalling pathways, the changes in Ca^2+^ levels observed in insect hemocytes might suggest the presence of a similar mechanism in insect cells during fungal infection. The increased intracellular calcium concentration present in the *G. mellonella* hemocytes during fungal infection might be a crucial factor in apoptosis induction and, as a result, the death of immunocompetent cells. However, there are no research data about the role of mitochondrial Ca^2+^ influx in insect cell death. The increase of the cytosolic free calcium ([Ca^2+^]i) concentration was observed in the *S. litura* cell line (SL-1) after infection by the *Syngrapha falcifera* multiple nuclear polyhedrosis virus (SfaMNPV); however, in that case, neither the elevation of the cytosolic calcium ion nor extracellular calcium entry was the inducing factor of apoptosis, which hinted that the depletion of the ER Ca^2+^ store contributed to SL-1 cell apoptosis induced by SfaMNPV [62]. In *Drosophila melanogaster* mitochondrial Ca^2+^ stores are critical for signal amplification olfactory sensory neurons [63].

In eukaryotes, most ATP is synthesized by mitochondrial oxidative phosphorylation, during which several reactive oxygen species (ROS) are produced [64]. The overload of mitochondrial Ca^2+^ leads to the increased generation of ROS and decreased ATP production in mammals. Previous research indicates that oxidative stress plays an important role in *G. mellonella* hemocyte destruction during *C. coronatus* infection; Kazek et al., report the presence of an increased level of 8-hydroxy-2′-deoxyguanosine (8-OHdG), a biomarker of oxidative DNA damage in fungus-treated hemocytes together with lowered levels of antioxidant enzymes, including superoxide dismutase (SOD), catalase (CAT), and glutathione peroxidase (GPx) [26].

Our present findings indicate several disturbances in ATP production, and its use as a substrate for aerobic respiration, in insect mitochondria during fungal infection. More specifically, an increase in the ADP/ATP ratio was noted, as well as a decrease in ATP-coupled oxygen consumption and an increase in non-ATP-coupled oxygen consumption in insect hemocytes (Figure 1D and Figure 4E). The elevated level of non-ATP-oxygen consumption indicates higher proteon leaks in mitochondria and, thus, ROS generation, which might be a principal source of oxidative stress and cell death in hemocytes. The high ADP/ATP ratio results in decreased levels of ATP and increased levels of ADP, which is characteristic of apoptotic or necrotic cells. All disturbances in ATP production and metabolism result in higher ROS production and, hence, oxidative stress, triggering programmed cell death in *G. mellonella* hemocytes during fungal infection.

To compensate for the decrease in ATP production resulting from mitochondrial toxicity, oxygen consumption is decreased, and glycolytic flux is increased (extracellular acidification). Our present findings confirm the presence of disturbances in ATP production due to changing oxygen consumption in the *G. mellonella* hemocytes during fungal infection (Figure 4A–C). *C. coronatus* treatment decreased both intracellular and extracellular oxygen consumption, and at the same time increased extracellular acidification in *G. mellonella* hemocytes. Elevated extracellular acidification is a marker of increased anaerobic respiration; it has been shown that mitochondrial toxins (electron transport inhibitors) prevent or restrict aerobic ATP generation by oxygen consumption. To compensate for this loss of cellular ATP, cells respond by increasing their glycolytic activity which directly results in an increase in acidification. All these changes were observed in insect hemocytes during fungal infection, which indicate a failure in mitochondria physiology, resulting in the activation of apoptosis and eventual cell death.

Mitochondrial dysfunction is caused by membrane depolarization [7]. The most important triggers for MPTP opening are Ca^2+^ and ROS; in living cells, these act in conjunction with a variety of pathological challenges [55]. Changes in the membrane potential, along with decreases in ATP to ADP ratios, increases in mitochondrial matrix calcium levels and oxidative stress, and the release of cytochrome c into the cytosol are all presumed to be associated with the mitochondrial permeability transition, resulting in disruption of ions and small molecule homeostasis via the MPTP [8]. Our findings confirm MPTP opening (Figure 3), and fluorescence microscopy revealed changes in membrane potential (Figure 2) in insect hemocytes during fungal infection. The activation of mitochondrial permeability transition pores (MPTPs) and the loss of mitochondrial membrane potential (MMP) were observed also very early during apoptosis in Sf9 cells after treatment with azadirachtin and camptothecin [20].

However, spectrofluorometric research did not reveal any similar changes or only found them in a lower percentage of cells. These differences may be due to the methods of sample preparation; the spectrofluorometric analysis was based on freshly collected full hemolymph, containing all classes of hemocytes, and fluorescent analysis on cultured hemocytes, where granulocytes and plasmatocytes predominate. These two classes of immunocompetent cells are the only hemocytes adhering to foreign bodies, which enables them to participate in phagocytosis, encapsulation, and nodulation. Our results suggest that the described changes in mitochondria morphology are characteristic only for these two classes. These play an essential role in insect immunological defence, and, hence, our findings highlight the crucial impact of mitochondrial disturbance in hemocyte death during fungal infection.

This thesis is supported partly by a flow cytometry analysis, which indicated that separate populations of *G. mellonella* hemocytes reacted differently to *C. coronatus* infection. The analysis showed the presence of two main populations of wax-moth immunocompetent cells in control insects, and that fungal infection caused a greater amount of cell death and changes in mitochondrial membrane potential in one of them (Figure 2C); it is possible that this population contains plasmacytes and granulocytes. However, there is no literature data describing the method of hemocyte sorting and, for that reason, there is a need to conduct further research focused on checking the apoptotic changes in the two isolated populations. Moreover, the result of analysis of fungus-treated larvae with no sign of infection is similar to the control ones, which might point to the significant role of mitochondrial membrane potential disturbance in hemocyte death during fungal infection.

The different reactions of the individual classes of hemocyte might account for the statistically insignificant differences observed between the groups in changes in respiratory rate and mitochondrial capacity during fungal infection. Although these parameters have been found to decrease during fungal infection, the changes were not significant. These results also might suggest that different kinds of hemocytes react differently to fungal infection.

However, a significant decrease in ATP-coupled oxygen consumption was noted, as well as a significant increase in non-ATP oxygen consumption; these findings suggest that disturbances in ATP production and aerobic respiration might be a more universal way of hemocyte destruction and might serve as important virulence factors of entomopathogenic fungi or as insect defence mechanisms; the death of cells infected by fungus might be an essential defence mechanism.

MPTP opening induces mitochondrial swelling. These large-scale alterations of organelle morphology and mitochondrial membrane permeabilization allow the release of proapoptotic factors, such as cytochrome c, into the cytosol. As a soluble protein, cytochrome c is localized in the intermembrane space and is loosely attached to the surface of the inner mitochondrial membrane [21]. Its release from mitochondria is a key event and plays an important role in initiating apoptosis in the mammalian cell [65]; however, the role of cytochrome c is not clear in insect-cell apoptosis. There are contradictory reports on the role of cytochrome c in apoptosis in *Drosophila* following its release from mitochondria [66,67]. In Lepidoptera, the literature data suggest that cytochrome c plays an essential role during apoptosis (e.g., *Spodoptera frugiperda*, *Spodoptera exigua*, *S. litura*, and *B. mori*) [18,19,21,23,62,68,69,70,71,72]. On the other hand, the silencing of the expression of cytochrome c had a remarkable effect on procaspase-3 and procaspase-9 activation and resulted in the reduction of caspase-3 and caspase-9 activity in Sl-1 cells undergoing apoptosis [21]. Our present findings confirm the presence of a cytochrome c-like protein in the cytosol fraction in *G. mellonella* hemocytes after fungal infection, which might suggest the transition of this protein from mitochondria. This translocation is probably a result of the mitochondrial dysfunction described above, as indicated by literature confirming the presence of MPTP-dependent cytochrome c release mechanisms in lepidopteran cells [20]. However, more data are needed to confirm this thesis.

## 4. Materials and Methods

### 4.1. Insects

A culture of the wax moth, *G. mellonella,* was maintained and reared in temperature and humidity-controlled chambers (30 °C, 70% r.h.) in constant darkness on an artificial diet [73]. Fully-grown larvae were collected before pupation, surface-sterilized, and homogenized, and then used as a supplement in the fungal cultures. Five-day-old last instar larvae were used to analyse the influence of fungal infection on the morphology and functioning of mitochondria in insect hemocytes.

### 4.2. Fungus

*C. coronatus* (isolate number 3491), originally isolated from *Dendrolaelaps* spp., was obtained from the collection of Prof. Bałazy (Polish Academy of Sciences, Research Center for Agricultural and Forest Environment, Poznań, Poland). It was routinely maintained in 90 mm Petri dishes at 20 °C with cyclic changes of light (L:D 12:12) on Sabouraud agar medium (SAM) with the addition of homogenized *G. mellonella* larvae to a final concentration of 10% wet weight. The sporulation and virulence of the SAM *C. coronatus* cultures were enhanced with the addition of homogenized *G. mellonella* larvae.

### 4.3. Infection of Insects with C. coronatus

*G. mellonella* larvae (five-day-old last instar) were exposed for 24 h at a temperature of 20 °C to fully-grown and sporulating *C. coronatus* colonies. Fifteen individuals were maintained in each Petri dish. A control group was formed of larvae exposed for 24 h to sterile Sabouraud agar medium (Merck Millipore, Darmstadt, Germany). After exposure, the insects were transferred to new, clean Petri dishes on an artificial diet [73], and kept at 20 °C for one day. Following this 24-h exposure to the fungus, one group of insects was collected immediately for examination (F24 group) while the rest were left for another 24 h before collection (F48 group).

### 4.4. Larval Hemolymph Collection

*G. mellonella* hemolymph was collected from both the control and infected (F24 and F48) larvae. Before bleeding, the insects were disinfected in 70% ethanol, and immersed in distilled water to reduce the contamination of hemolymph samples. Hemolymph was taken from the larvae through an incision made in the last proleg. The hemolymph was prepared in different ways depending on the planned method.

For hemocyte culture, 100 μL of fresh hemolymph collected from ten larvae were suspended in 500 μL of supplemented Grace’s Insect Medium (GIM; Invitrogen, Thermofisher, Carlsbad, CA, USA) with added gentamycin (10 mg/mL; Gibco, Life Technologies, Carlsbad, CA, USA), amphotericin B (250 μg/mL; Gibco, Life Technologies, Carlsbad, CA, USA), and phenylothiourea (PTU; 0.1 mM; Sigma Aldrich, München, Germany). It was then transferred to a six-channel μ-Slide IV 0.4 (IBIDI, Martinsried, Germany)—100 μL for each channel. The slides were incubated at 27 °C for 24 h.

For spectrofluorometric analysis, 20 drops of fresh hemolymph collected from 20 larvae were suspended in 100 μL of phosphate-buffered saline (PBS) with PTU on ice. This suspension was used to determine the ADP/ATP ratio. For other analyses, the hemolymph was centrifuged (300× *g*, 4 °C, 5 min), and the hemocyte pellet was taken for further testing.

For flow cytometric analysis, 100 μL of fresh hemolymph collected from ten larvae were suspended in 100 μL of supplemented GIM with 10 mM EDTA (ethylenediaminetetraacetic acid) and 30 mM sodium citrate. The hemolymph was centrifuged at 300× *g* at room temperature (5 min) and the pellet was collected for future analysis.

### 4.5. Staining of Mitochondria in Fluorescent Microscopy

The hemocyte cell cultures were prepared according to Section 2.4. After 24 h of incubation, the cells were fixed in 4% paraformaldehyde (Sigma Aldrich, München, Germany; PFA) in phosphate-buffered saline (PBS) and permeabilized in 0.1% Triton X-100 (Sigma Aldrich, München, Germany) in PBS. Mitochondria were detected with a MITO-ID Red Detection Kit (Enzo Life Sciences, Farmingdale, NY, USA). The cells were incubated for 30 min with Dual Detection Reagent and ActinGreen 488 ReadyProbes Reagent (Invitrogen, Thermofisher, Carlsbad, CA, USA) was used to label the actin fibres. The cell nuclei were stained with Hoechst (Enzo Life Sciences). Fluorescence signals were analysed by fluorescent microscopy using an Axio Vert.A1 fluorescence microscope (Carl Zeiss, Jena, Germany) with Axio Cam ICc 5 (Carl Zeiss, Jena, Germany).

### 4.6. The Calculation of Ca^2+^ Concentration in Hemocytes

The changes in Ca^2+^ concentration in hemocytes were detected by spectrofluorometric analysis using the FLUOFORTE Calcium Assay Kit (Enzo Life Sciences).

The hemocytes, prepared as above, were resuspended in FLUOFORTE Dye-Loading Solution and then plated to a black 96-well plate (nest) in a plating volume of 100 µL. The cell suspensions were then incubated for one hour at room temperature. The calcium flux was monitored as the fluorescence insensitivity at Ex = 490 nm and Em = 525 nm, measured using a Synergy HT Microplate Reader (BioTek, Instruments, Inc., Winooski, VT, USA) every 10 min for 50 min. The test was conducted as three independent replicates.

### 4.7. The Detection of Changes in ADP/ATP Ratio

To detect changes in the ADP/ATP ratio, the ADP/ATP Ratio Assay Kit (Sigma Aldrich, München, Germany) was used. Briefly, 10 µL amounts of freshly prepared hemolymph were plated in a white 96-well plate (CytoGen, Zgierz, Poland) and ATP reagent was then added to each well. The luminescence was read after one-minute incubation at room temperature (*RLU_A_*). The plate was incubated for another 10 min, and the luminescence was read again (*RLU_B_*). Immediately afterwards, the ADP reagent was added to each well and mixed by tapping the plate or pipetting. After another minute, the luminescence (*RLU_C_*) was read. The ADP/ATP ratio was calculated as:

$$\mathrm{ADP}/\mathrm{ATP}\;\mathrm{ratio}=\frac{RLU_{C}-RLU_{B}}{RLU_{A}}$$

### 4.8. The Measurement of Protein Concentration—The Bicinchoninic Acid Assay (BCA Method)

Protein concentration was measured using the commercial Pierce BCA Protein Assay Kit (Thermo Fisher Scientific, Waltham, MA, USA). Briefly, the samples were prediluted 100Xin PBS (this dilution was included in the final calculations) and 25 µL of each dilution were transferred to a 96-well plate (NEST, Wuxi, China); following this, 200 µL of “working reagent”, prepared according to the manufacturer’s instructions, was added to each well. The samples were incubated at 37 °C for 30 min. After this time, the plate was cooled to room temperature and the absorbance was read at 562 nm in a BioTek HT spectrofluorometer. The protein concentration in the tested samples was measured based on a calibration curve. Each sample was prepared in three replications.

### 4.9. Measurement of Caspase Activity

Caspase-9-like protein activity was measured using the commercial Caspase-9 colourimetric assay kit (Enzo Life Sciences, Farmingdale, NY, USA) according to the manufacturer’s protocol. Briefly, the samples were collected as described above (Section 2.4). The haemocyte pellets were dissolved in cell lysis buffer, incubated for 10 min on ice and then centrifuged (10,000× *g*, 4 °C, 1 min). The protein content was measured in the supernatant using the BCA method. From each sample, 200 μg of protein (experimentally selected amount) were added to separate wells in a 96-well plate (Nest, Wuxi, China), then mixed with 50 µL of 2X reaction buffer (containing 10 mM DTT, dithiothreitol) and 5 µL of 200 µM substrate (4 mM LEHD-pNA). The samples were incubated at 37 °C for two hours. After this time, the absorbance was read at 400 nm. Fold increase in caspase activity was determined by comparing the level of infected larvae and the noninfected control.

### 4.10. Detection of the Changes in Membrane Potential in G. mellonella Hemocyte Mitochondria

The changes in the membrane potential were determined with the MITO-ID^®^ membrane potential detection kit and MITO-ID^®^ membrane potential cytotoxicity kit (both Enzo Life Sciences, Farmingdale, NY, USA). As a positive control, the carbonyl cyanide 3-chlorophenylhydrazone (CCCP) was used.

Changes in membrane potential were determined by fluorescent microscopy and flow cytometry using hemocytes from the control and infected insects, prepared as described above. Hemolymph from fungus-treated insects with no sign of infection was used additionally for flow cytometry. To the positive control, 1 µL of 200 µM CCCP was added 15 min before the experiment and incubated at 27 °C. The controls and infected cells were washed in a washing buffer and then incubated with the dual detection reagent for 15 min at room temperature; the samples were protected from light. Fluorescence signals were analysed by fluorescent microscopy using an Axio Vert.A1 fluorescence microscope (Zeiss) with Axio Cam ICc 5 (Zeiss) and by flow cytometry using a CyFlow Cube 8 (Sysmex, Norderstedt, Germany). The readings were analysed with FCS Express 7 (DeNovo Software).

The changes in membrane potential were detected by spectrofluorometry. The hemocytes were resuspended in MITO-ID^®^ MP Dye Loading Solution, and then plated in a 96-well dark plate (Sigma Aldrich, München, Germany), at a volume of 100 µL per well (in three replicates). The positive control was treated with 1 µL of 200 µM CCCP 15 min before the experiment and incubated at 27 °C. The membrane potential was monitored as the fluorescence insensitivity at Ex = 490 nm and Em = 590 nm using a Synergy HT Microplate Reader (BioTek) every 10 min for 90 min.

### 4.11. Detection of Mitochondrial Permeability Transition Pore (MPTP) Opening

MPTP opening was detected by flow cytometry using the Mitochondrial Permeability Transition Pore Assay Kit (Abcam, Cambridge, UK).

For each sample, 1 mL aliquots of hemocyte suspension were added to four separate tubes using MPTP Wash Buffer: one tube did not receive any treatment (tube 1), one tube with MPTP Staining Dye only (1:500 in wash buffer; tube 2), one tube with MPTP staining dye and cobalt (II) chloride (5 µL of CoCl_2_; tube 3) and one tube with MPTP staining dye, CoCl_2_ and Ionomycin (5 µL of Ionomycin; tube 4).

The samples were then incubated at 37 °C for one hour, protected from light. After incubation, the cells were pelleted by centrifugation at 1000× *g* for 5 min in RT and then resuspended in 1 mL of wash buffer to remove excess staining and quenching reagents. After staining, the cells were kept on ice and analyzed within one hour by flow cytometry on a CyFlow Cube 8 (Sysmex, Norderstedt, Germany) and analysed with FCS Express 7 (DeNovo Software).

### 4.12. The Detection of Changes in Oxygen Consumption

Any changes in oxygen consumption were detected using MITO-ID Extracellular O_2_ Sensor Kit (High Sensitivity), MITO-ID Intracellular O_2_ Sensor Probe, MITO-ID (both Enzo Life Sciences, Farmingdale, NY, USA), Mitochondrial Stress Test Complete Assay Kit (Abcam, Cambridge, UK) and Extracellular pH Sensor Kit (Enzo Life Sciences). Then, all changes were monitored as the fluorescence intensity at Ex = 380 nm and Em = 650 nm, with time resolve mode: delay time 30 μs and integration time 30 μs, measured using a Synergy HT Microplate Reader (BioTek) every 10 min for 90 min. Each experiment was conducted as three independent replicates.

To detect changes in extracellular oxygen consumption and glycolytic flux (extracellular pH changes) in cells, hemocytes (preparation described above—Section 2.4) were resuspended in PBS to measure extracellular oxygen consumption; to detect pH changes, they were placed in respiration buffer (1 mM K-phosphate, 20 mM glucose, 0.07 M NaCl, 0.05 M KCl, 0.8 mM MgSO_4_, 2.4 mM CaCl_2_, and pH = 7.4), mixed with oxygen or pH extracellular sensor probe. The suspensions were then plated in a black 96-well plate (Sigma Aldrich, München, Germany) to a volume of 100 µL. Following this, one drop of mineral oil (Enzo Life Science) was added to each well and the result was immediately read on a fluorescence plate reader.

To detect changes in intracellular oxygen consumption, hemocytes were cultured in a black 96-well sterile plate for 24 h, as described in Section 2.4. Following this, 10 µL of intracellular sensor probe were added and the cells were incubated for another 24 h and then read on a fluorescence plate reader.

Real-time analysis of cellular respiration and mitochondrial function was performed using the Mitochondrial Stress Test Complete Assay Kit (Abcam, Cambridge, UK). Briefly, the hemocytes were resuspended in a 96-well plate (Nest) to reach a concentration of ~4 × 10^5^ cells in a 90 μL medium (GIM). Following this, 10 μL of the extracellular O_2_ probe were added to each well, apart from the wells used as blank controls. Next, to wells was added: 10 μL of oligomycin working stock (1.5 µM final concentration/well to measure ATP-coupled respiration), 10 μL of carbonyl cyanide-p-trifluoromethoxyphenylhydrazone working stock (FCCP, 2.5 µM final concentration/well to measure maximal respiration), 10 μL of antimycin A working stock (1 µM final concentration/well, to measure nonrespiratory oxygen consumption), or 10 μL reconstituted glucose oxidase (1.5 mg/mL) to the signal control wells, respectively. Each well was overlaid with two drops of HS mineral oil and immediately read on a fluorescence plate reader.

### 4.13. Isolation of Cytochrome c-like Protein from G. mellonella Larvae’ Hemolymph

Cytochrome c-like protein was isolated from the hemolymph of infected and noninfected *G. mellonella* larvae using a commercial isolation kit (Cytochrome c releasing apoptosis assay kit, Enzo Life Sciences, Farmingdale, NY, USA). The samples were collected from 100 larvae for each variant, as described in Section 2.4. Then, the hemolymph was centrifuged (600× *g* for 5 min. at 4 °C); the pellets were resuspended with 1 mL of 1Xcytosol extraction buffer mix containing DTT and Protease Inhibitors and then incubated on ice for 10 min. The samples were then homogenized in an ice-cold Kimble dounce tissue grinder (Merck Millipore, Darmstadt, Germany) (30 passes) and the homogenates were transferred to a fresh 1.5 mL tube and centrifuged at 700× *g* for 10 min. at 4 °C. The supernatants were transferred again to a fresh 1.5 mL tube and centrifuged at 10,000× *g* for 30 min at 4 °C. The supernatants were collected as a cytosolic fraction and pellets were resuspended in 0.1 mL of the mitochondrial extraction buffer mix containing DTT and protease inhibitors, vortexed for 10 s and saved as the mitochondrial fraction. After isolation, the protein content of both fractions was checked using the BCA method (as described above) and frozen at −80 °C.

### 4.14. Detection of Cytochrome c-like Protein in the Mitochondrial and Cytosolic Fractions (Western Blot Method)

An equal number of proteins (30 μg) from both treated and untreated cells were loaded and electrophoresed on 4–20% SDS-polyacrylamide gels (Mini-Protean Gels, 4–20%, Bio-Rad Laboratories Inc., Hercules, CA, USA). At the end of the electrophoresis, the proteins were blotted onto the nitrocellulose membrane (0.45 µm, Thermo Fisher, Carlsbad, CA, USA). The efficiency of the process was checked using a commercial membrane stain kit (MemCode Reversible Protein Stain Kit, Thermo Fisher, Carlsbad, CA, USA). Then the membrane was dried and preblocked with 5% skimmed milk prior to incubation with the primary antibodies: 1 μg/mL, murine monoclonal antibody, attached to the Cytochrome c releasing apoptosis kit assay kit (Enzo Life Sciences). The membrane was then probed with secondary antimouse antibodies conjugated to horseradish peroxidase (final antibody concentration—0.13 µg/mL, Enzo Life Sciences, Farmingdale, NY, USA). The immunoreactions between the antibodies were detected by the Covalight Detection System-enhanced chemiluminescence apparatus (Enzo Life Sciences) and recorded using the ChemiDoc MP gel visualization system (Bio-Rad).

### 4.15. Statistical Analysis

All data were expressed as mean ± standard deviation. The normality of their distribution was checked using the Kolmogorov–Smirnov (K-S) test. The ANOVA test and Tukey’s HSD Test were used to compare them. The significance level was assumed to be 95% (*p* < 0.05). STATISTICA 6.1 software (StatSoft Polska, Kraków, Poland) was used for all statistical testing.

## 5. Conclusions

The research presented in this paper indicates that during fungal infection, several changes in mitochondrial physiology and morphology take place in *G. mellonella* hemocytes and that these disturbances accompany apoptosis in insect immunocompetent cells. Most of them are similar to changes occurring in mammalian cells, which would reflect the considerable evolutionary conservatism regarding the role of mitochondria in apoptosis activation in insects and mammals. However, further research is needed to determine the presence, activation, and similarity of particular proteins taking part in the intrinsic pathway of apoptosis activation in mammals and insects during fungal infection, and to understand the changes of concentration that take place. Taking into consideration these similarities between insects and mammals, the use of *G. mellonella* as a model organism in preliminary studies clearly merits further consideration and evaluation.

## Figures and Tables

**Table 1:** The changes in concentration of mitochondrial and cytosol fraction in *G. mellonella* hemocytes after fungal infection.

Fraction	Sample	Protein Concentration [µg/µL]
mitochondrial	control (Cm)	8.41
	F24m	7.76
	F48m	6.97
cytosol	control (Cc)	4.18
	F24c	7.76
	F48c	4.78

F24 larvae sampled immediately after 24-h exposure to fungal infection; F48 larvae sampled 24 h after 24-h exposure; m—mitochondrial fraction; c—cytosol fraction.

## Data Availability

All data generated or analyzed during this study are included in this published article.
